# Effect of Silicone Oil on Properties and Performance of Ceramizable Styrene-Butadiene Rubber-Based Composites

**DOI:** 10.3390/polym15153204

**Published:** 2023-07-28

**Authors:** Mateusz Imiela, Dariusz M. Bieliński, Magdalena Lipińska, Przemysław Rybiński

**Affiliations:** 1 Institute of Polymer & Dye Technology, Faculty of Chemistry, Lodz University of Technology, Stefanowskiego 16, 90-537 Łódź, Poland; dariusz.bielinski@p.lodz.pl (D.M.B.); magdalena.lipinska@p.lodz.pl (M.L.); 2 Institute of Chemistry, The Jan Kochanowski University, 25-406 Kielce, Poland; przemyslaw.rybinski@ujk.edu.pl

**Keywords:** polymer composites, ceramization, ceramification, styrene-butadiene rubber, silicone oil

## Abstract

New trends in the circular economy and sustainability are pointing towards the gradual elimination of standard flame retardants such as phosphorus compounds or halogenated compounds. New solutions are therefore being sought in this area and ceramizable composites could be an interesting alternative. Weak rheological properties are one of the main disadvantages of ceramizable composites. This study tested ceramizable composites composed of styrene-butadiene rubber (SBR) as a polymer matrix and mica as a mineral filler and aimed to improve the viscoelastic properties of silicone oil as a plasticizer. To characterize this composite’s mechanical properties before and after ceramization, the viscoelastic properties were tested with a dynamic oscillating rheometer and the thermal behavior with a cone calorimeter. This paper also provides results showing differences (via the abovementioned properties) between vulcanization with sulfur and that with peroxide for the ceramizable composites based on SBR. The presented results, showing changes in mechanical properties, dynamic viscosity or flammability, among others, allow a better understanding of elastomeric composites with ceramizable flame-retardant systems. Such composites can find a wide range of applications, from lagging for electrical cables to building elements such as floor coverings and fire barriers.

## 1. Introduction

One of the most unique flame-retardant systems for polymer materials, characterized by a physical mechanism of action, is ceramization [1,2,3,4,5,6,7,8]. In conditions of elevated temperatures and/or fire, ceramizable composites initiate the process of formation of a ceramic structure on the surface of the composite, with very good barrier and mechanical properties. The resulting layer reduces the diffusion of flammable volatile substances to the burning area and the oxide to the bulk of the composite, decreasing the combustion kinetics. The ceramic structure causes also the inhibition of heat transport into the bulk of the material, which slows down the process of the thermal destruction of the polymer, and thus the rate of fuel production [9,10,11,12,13].

**Figure 1:**
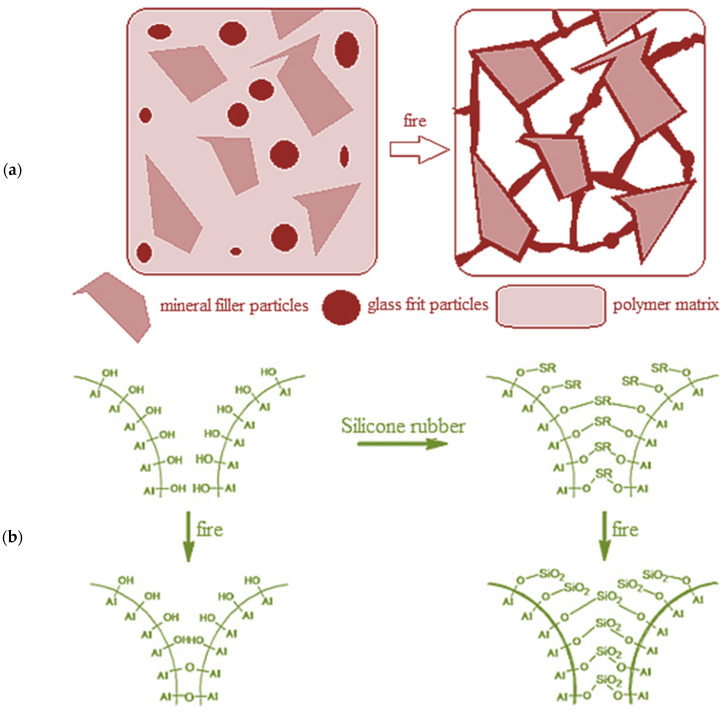

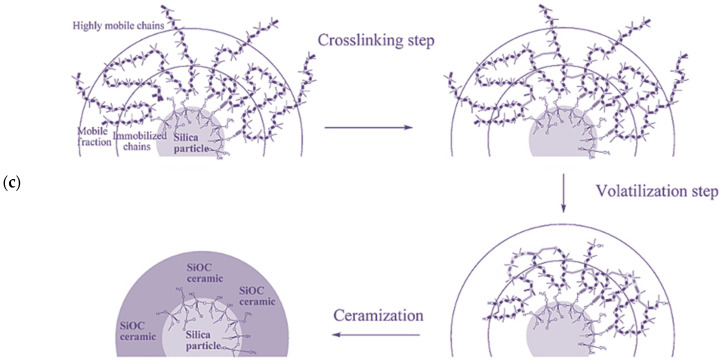
Different models of ceramization process: (**a**) with the fluxing agent; (**b**) with the creation of SiO_2_ particles from SR between mineral filler particles; (**c**) with the creation of SiOC ceramic on Silica particles [11,12,13]. Reproduced with permission from Xin-Hao Gong, Tao-Yuan Wu, Jie Ma, Dong Zhao, Yu-Cai Shen, Ting-Wei Wang, J. Alloys Compd., 2017.

The ceramic structure on the surface of a ceramizable composite material can be formed in several ways: (1) the application of glass frits with a relatively low softening point temperature, which improves the efficiency of ceramization by the formation of additional physical adhesive bridges between the thermally stable mineral filler particles—this allows us to apply of the effect of ceramization in dispersion composites, in which the continuous phase is a fully organic polymer, unable to form silica during thermal destruction (Figure 1a) [14,15]; (2) the formation of silica bridges between the mineral filler particles during the thermooxidation and degradation of the silicone matrix (Figure 1b) [2,16]; (3) the sintering of mineral particles via the condensation of the hydroxyl groups present on their surfaces (Figure 1b) [17]; (4) the formation of a ceramic phase in the form of silicon oxycarbide (SiOC) as a result of the crosslinking and ceramization of the silicone matrix. It has been proven that the addition of a platinum catalyst or active silica increases the efficiency of the crosslinking process (Figure 1c) [5].

This research aims to develop a novel styrene-butadiene rubber-based ceramizable composite with enhanced viscoelastic properties via the application of silicone oil reducing filler/filler and filler/elastomer interactions simultaneously, actively taking part in the ceramization process. Elastomeric ceramizable composites, for the most part, consist of fillers that promote ceramizable flame retardancy. Typically, these are thermally stable mineral fillers (such as mica, wollastonite, talc, kaolin) and a fluxing agent, which can be a mixture of various metal oxides characterized by relatively low softening temperatures (approximately 400 °C). These fillers allow the formation of a barrier that blocks fire propagation without releasing toxic substances into the atmosphere. Due to their advantages and, in particular, their physical flame-retardant methods, cermizable elastomeric composites may be an interesting alternative to current flame-retardant systems. Taking into account global trends related to the circular economy and sustainability, materials of this type could be applied in many industries in the future.

## 2. Materials and Methods

### 2.1. Materials

The following raw materials were used to produce the elastomer composites.

- Elastomer base—synthesized by emulsion method with styrene-butadiene rubber (KER 1500, Synthos S.A., Oswiecim, Poland). The properties of this elastomer are as follows: Monney viscosity (ML 1 + 4; 100 °C)—45–55 ML, bonded styrene—22–25%, organic acids—5.0–7.5%, volatile matter—max. 0.7%, soap—max. 0.4%, total ash—max. 0.4%.
- Crosslinking agents—2,4-dichlorobenzoyl peroxide (50% paste) or sulfur. For sulfur vulcanization, we used N-cyclohexyl-2-benzothiazole sulfenamide (CBS) as an accelerator and stearic acid and zinc oxide as activators.
- Antioxidant—2,2,4-trimethyl-1,2-dihydroquinoline (TMQ).
- Mineral filler—mica phlogopite (PW30, LKAB Minerals GmbH, Lulea, Sweden) with a specific surface area of 2.8 m^2^/g.
- Fluxing agent—chemical composition of metal oxides (wt.%): 4 Li_2_O, 16 Na_2_O, 37 B_2_O_3_, 43 SiO_2_ (A 4015, Reimbold & Strick GmbH, Cologne, Germany), with softening point temperature of 540 °C.
- Plasticizer—silicone oil (Silikony Polskie Sp. z o. o., Nowa Sarzyna, Poland).

### 2.2. Preparation of Rubber Samples

**Figure 2:**
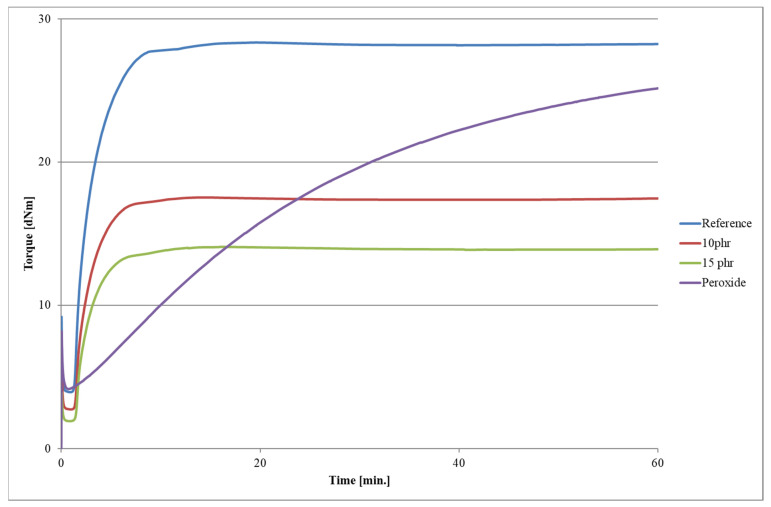
Rheometric curves from vulcanization for the ceramizable composites.

The composite mixes (Table 1A) were prepared with a laboratory two-roll mill (length of the rolls—200 mm; diameter—150 mm; Bridge, UK), working with a rotation speed in the slower roll of 18 rpm (revolutions per minute) and in the faster roll of 20 rpm (friction—1.1). The kinetics of vulcanization of the elastomer mixes were tested through an Alpha Technologies MDR2000 rheometer (Alpha Technologies, Wilmington, DE, USA) according to PN-ISO 37:1998 [18]. According to the obtained results (Table 1B and Figure 2), the samples were formed and vulcanized in steel molds by a laboratory press at 160 °C (sulfur-based curatives) or 130 °C (peroxide cured) and under 10 Mpa of pressure. The addition of silicone oil did not affect the nature of the vulcanization process. In all cases, a vulcanization plateau was clearly visible after the maximum torsional moment was reached. The situation was different when the sulfur crosslinking unit was replaced with dicumyl peroxide. Vulcanization then had a marching characteristic. Approaching 60 min, the curve started to flatten out; however, it was apparent that, with the use of dicumyl peroxide, this was an inefficient method of crosslinking styrene-butadiene rubber.

The sub-vulcanization and vulcanization times were much longer when peroxide was used. The values of the torsional moments, however, did not deviate as significantly compared to the reference sample. The addition of the oil reduced the magnitude of the torsional moments and resulted in a shorter vulcanization time.

### 2.3. Experimental Techniques

Viscoelastic properties were measured using a MonTech RPA 3000 rheometer in accordance with ASTM D6204 Part A for low strain and Part B for high strain. These measurements were performed with increasing frequencies of 0.1 Hz, 0.2 Hz, 0.4 Hz, 0.6 Hz, 0.8 Hz, 1 Hz, 2 Hz, 5 Hz, 10 Hz, 15 Hz, 20 Hz, 30 Hz, 40 Hz and 50 Hz (10 cycles for each frequency) twice for each composite, low strain 7% and high strain 100%, at a temperature of 100 °C. For the third viscoelasticity measurement, the samples were first vulcanized in a rheometer at 160 °C, and then cooled to 100 °C and tested at a constant frequency of 10 Hz, while the strain amplitude varied at 0.1%, 0.2%, 0.4%, 0.6%, 0.8%, 1%, 2%, 4%, 6%, 8%, 10%, 20%, 40%, 60%, 80% and 90% (10 cycles for each strain).

The mechanical properties of the vulcanizates were tested with a Zwick/Roell 1435 testing machine and Zwick/Roell hardness tester, Germany.

The combustibility of the vulcanizates was determined using a cone calorimeter (Fire Testing Technology Ltd., East Grinstead, UK). Horizontally in relation to the IR heating source of 35 kW/m^2^, samples with dimensions 100 mm × 100 mm × 2 mm were placed.

Ceramization of the vulcanizates was performed in a laboratory furnace, FCF 2.5SM (Czylok, Poland). Cylindrical samples (diameter—16 mm, height—8 mm) of the composites were heated in 3 different conditions: (1) 1100 °C—from room temperature to 1100 °C in 30 min (heating rate 35 $\frac{{}^{\circ}C}{min}$), (2) 950 °C—from room temperature to 950 °C in 120 min (heating rate 7.5 $\frac{{}^{\circ}C}{min}$), (3) 550–1000 °C—from room temperature to 550 °C in 53 min (heating rate 10 $\frac{{}^{\circ}C}{min}$), 10 min of isothermal conditions at 550 °C and at the end heating from 550 °C to 1000 °C in 27 min (heating rate 16 $\frac{{}^{\circ}C}{min}$)—total time 90 min.

## 3. Results and Discussion

### 3.1. Viscoelastic Behavior of Composites

The complex dynamic viscosity η*, which is measured by an oscillating rheometer, is analogous to viscosity η_app_ measured by a capillary rheometer [19,20,21,22]. The complex dynamic viscosity η* is calculated from parameters obtained from a dynamic oscillating rheometer (RPA) as follows:

(a) the complex shear modulus G* is equal to $G^{*}=\sqrt{{\left(G^{\prime }\right)}^{2}+{\left(G^{\prime \prime }\right)}^{2}}$, where G′ is the storage shear modulus and G″ is the loss shear modulus;
(b) the complex dynamic viscosity η* is equal to $\eta *=\frac{G*}{\omega }$; ω—frequency (Hz).

Shear stress is needed to break off the structure of uncrosslinked rubber and to destroy the network of filler aggregates. The deformation of samples using high and low stress affects the value of the dynamic viscosity.

**Figure 3:**
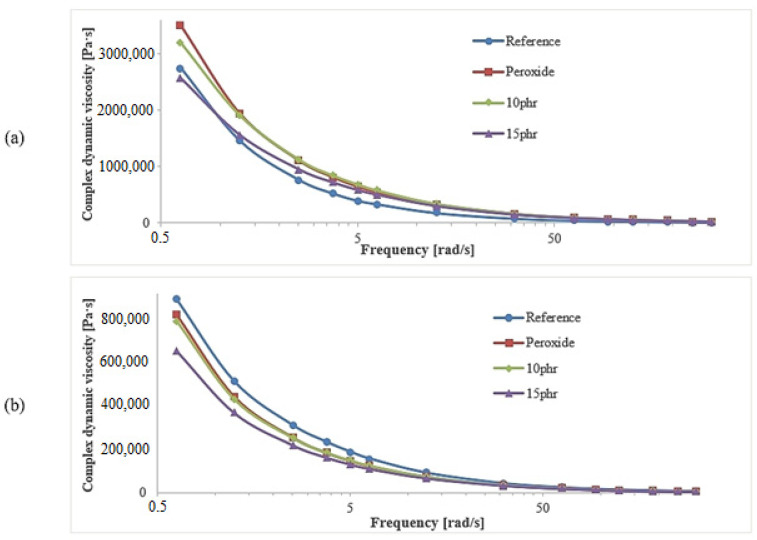
RPA dynamic complex viscosity versus frequency for the ceramizable composites: low strain 7% (**a**), high strain 100% (**b**).

The dynamic viscosity at low strain (Figure 3a) was four times greater than the viscosity at high strain (Figure 3b). In both cases, the lowest value of dynamic viscosity was reached for the composite filled with 15 phr of silicone oil. One can see from the obtained curves that the decrease in viscosity as a function of the frequency caused by both large and small rotor deflection, for most composites, was similar.

A greater thixotropic effect (dependence of viscosity on the time of shear force) is invisible in the case of harder/stiffer composites and, for them, higher energy and longer times are required to break the bonds within the filler network [23]. It is possible, therefore, that the reference and peroxide samples with the highest hardness experienced the largest viscosity decrease, while the least hard 10 phr and 15 phr had the smallest viscosity decrease, especially when we used higher shear forces.

Rubber is a viscoelastic material in both vulcanized and non-vulcanized forms. The elasticity of unvulcanized rubber is largely due to the entanglement of the polymer chains. A higher value of the elastic modulus G′ suggests a greater elastic response of the material at a given frequency, tension and temperature. This higher value of the elastic response indicates how well the polymer combines with the filler particles during mixing (how well the polymer occludes the filler particles). After adding silicone oil, G′ was more than two times higher than that of the reference sample (Table 2). From the low-strain curves, it can be seen that G′ increased in samples with 10 phr of silicone oil and decreased in samples with 15 phr of silicone oil, which could have been an effect of the saturation of compatibilization and led to plastification.

### 3.2. Mechanical Properties of Composites before Ceramization

**Figure 4:**
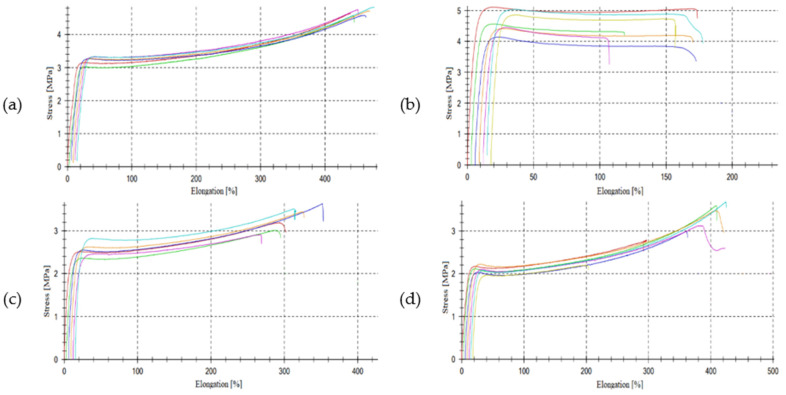
Stress–strain curves for ceramizable composites: reference (**a**), peroxide (**b**), 10 phr (**c**) and 15 phr (**d**). Each line describe different sample during test.

During the tear resistance and tensile strength tests before destruction, the delamination of the samples was visible. This effect was stronger in composites with silicone oil. Table 3 and Figure 4 show the mechanical properties of the composites studied.

With the growing addition of the silicone oil, the hardness of the composites decreased, which was in line with the results of the viscoelastic properties. The least flexible composite was the one crosslinked using peroxide. This result was expected because of the stiff nature of the carbon–carbon bonds formed by peroxide vulcanization. Such a crosslink morphology resulted in a significant reduction in the macroscopic elasticity of the composite. The addition of the silicone oil reduced the filler/filler and filler/polymer matrix interactions, which resulted in lower tensile strength and elongation at break values.

### 3.3. Combustibility

Good results in flammability tests such as cone calorimetry for ceramizable composites are a result of both the creation of a ceramic structure during exposure to fire or high temperatures and the dilution of the overall amount of combustible material present in the composite. The composites containing 300 phr of thermally stable mineral filler and glass frit of only 110–125 phr were derived from combustible materials such as polymers, some curative agents and, in this case, the plasticizer.

**Figure 5:**
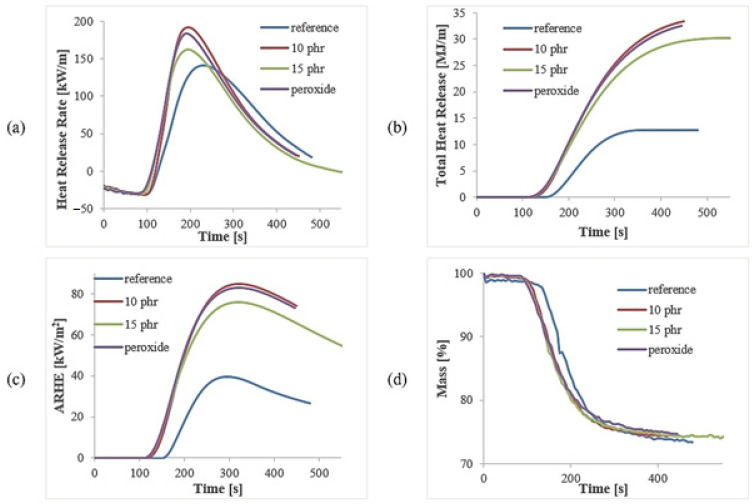
Cone calorimetry analysis of the composites: heat release rate (HRR) (**a**), total heat released (THR) (**b**), averaged heat release rate (ARHE) (**c**) and mass loss (**d**).

All modifications of the reference composite reduced the material’s flammability (Figure 5). Peroxide, 10 phr and 15 phr samples started to burn earlier and emitted more heat during combustion. This occurred because the sample was not dried in a vacuum dryer to remove the degradation products of peroxide and silicone oil. Because of this, in the first heating stage, these products evaporated, ignited and generated a first wave of heat, which further accelerated the degradation of the rubber. However, these results are still respectable in comparison with other ceramizable composites [24,25,26]. The addition of 15 phr of silicone oil resulted in a lower value of HRR and THR than the 10 phr sample. This may be a result of the greater impact of silica created during the decomposition of the silicone oil on the ceramic phase.

The values of the ratio of the maximum HRR peak (HRR_p_) and time to reach this peak (t_HRR_) are parameters that provide a great deal of information about the combustibility performance of a material. HRRp/tHRR decreased with the greater addition of silicone oil, which again confirms the hypothesis regarding the flame-retardant performance of the silica formed from the silicone oil’s thermal degradation when incorporated into the ceramic phase (Table 4). In this case, the much more thermally stable peroxide bond, created during vulcanization, between the macromolecules of the polymer is not relevant. The THR value of the peroxide composite, in comparison with the reference sample, was more than two-times higher. All the composites lost almost the same mass during burning.

### 3.4. Properties of Composites after Ceramization

**Figure 6:**
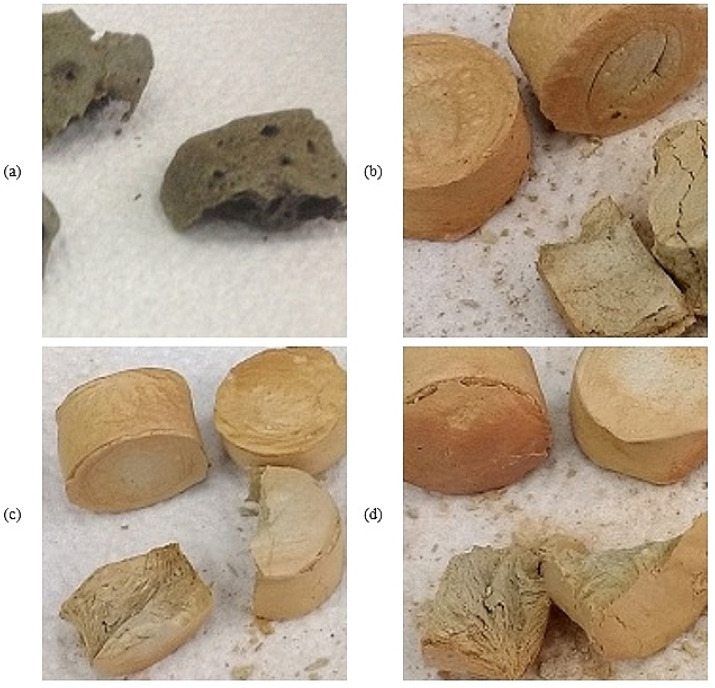
Appearance of the composites after ceramization and compression: reference (**a**), peroxide (**b**) 10 phr (**c**), 15 phr (**d**).

In every case, the samples had almost the same shape both before and after ceramization, even at a temperature 1100 °C, in which the heating rate was 35 $\frac{{}^{\circ}C}{min}$ (Figure 6).

The reference and peroxide composites exhibited almost the same level of compression strength (Table 5). The measured values were within the limits of statistical error, so there were no significant changes resulting from the type of curing agent used. The 10 phr and 15 phr composites were weaker in extreme conditions but much better in other ones. Some of the 15 phr samples reached a value of 500 N.

The good mechanical results of some of the ceramized samples filled with silicone oil may have resulted from the fact that silicone oil, during thermal decomposition, creates silica (in a similar way to silicone rubber), which can additionally improve the mechanical properties of the ceramic phase. This effect was especially visible in the appearance of the samples after ceramization. Only composites with silicone oil after ceramization to 1100 °C had the same shape as after vulcanization.

## 4. Conclusions

In some cases, silicon oil can improve the properties of ceramizable materials, such as their viscoelastic properties and the strength of the ceramic phase. This plasticizer greatly deteriorates the interactions between filler particles, resulting in lower dynamic viscosity. Moreover, during thermal decomposition, the creation of particles of silica can improve the mechanical properties of the ceramic structure. Unfortunately, both the mechanical and thermal properties were much worse than those of the reference sample. Differences between vulcanization with sulfur and that with peroxide were barely visible (compression strength after ceramization, dynamic viscosity) or favored the sulfur (thermal and mechanical properties). The appearance after ceramization was only better for peroxide composites, but this was the least important parameter.

The results in the presented manuscript may be relevant considering new trends related to the circular economy and sustainability. The likely changes and limitations in the use of the current standard flame retardants brought about by these trends necessitate a search for new solutions in this area. Elastomeric ceramizable composites may be an interesting alternative to “chemical” flame retardants due to their physical mechanisms of action.

Elastomeric ceramizable composites based on organic polymers should still be developed due to several of their properties, such as high viscosity, which should be improved before these materials are developed at a production scale.

## Figures and Tables

**Table 1:** Composition (in phr—parts per hundred parts of rubber) and vulcanization parameters of the ceramizable composite mixes at temperature of 160 °C for sulfur-based curatives (reference, 10 phr and 15 phr) and 130 °C for peroxide.

A	Composition			
Component	Reference	Peroxide	10 phr	15 phr
SBR	100	100	100	100
Mica	200	200	200	200
Glass frit	100	100	100	100
Curatives (sulfur-based)	10	-	10	10
Peroxide	-	1.5	-	-
Plasticizer	-	-	10	15
B	Vulcanization parameters			
Scorch time (t_05_)	2 min 30 s	3 min 0 s	1 min 45 s	1 min 45 s
Torque t_05_ (dNm)	4.93	5.19	3.48	2.30
Optimum curing time (t_90_)	19 min	44 min	5 min	5 min
Torque t_90_ (dNm)	28.35	23.07	16.02	12.74

**Table 2:** Viscoelastic properties of uncured composites at chosen frequencies: storage shear modulus (G′), loss shear modulus (G″) and complex dynamic viscosity (η*).

Name of the Composite	Frequency (rad/s)	Low Strain			High Strain		
		G′ (kPa)	G″ (kPa)	η* (Pa·s)	G′ (kPa)	G″ (kPa)	η* (Pa·s)
Reference	0.628	268.5	57.5	2746,188	62.9	60.9	875,638
	5.024	305.3	65.5	390,361	112.3	95.9	184,605
	94.2	412.1	109.9	28,435	174.0	156.2	15,585
	314	471.1	122.9	9738	192.0	148.7	4857
Peroxide	0.628	274.7	217.2	3,502,335	55.7	55.9	803,391
	5.024	413.6	305.3	642,537	80.9	82.3	144,289
	94.2	854.4	545.0	67,560	140.8	145.3	13,490
	314	1040.0	602.0	24,033	152.8	144.6	4208
10 phr	0.628	253.8	193.8	3,193,494	54.2	55.5	775,826
	5.024	455.4	291.9	676,193	84.8	80.8	146,383
	94.2	910.7	474.2	68,452	147.6	138.5	13,496
	314	1085.8	427.0	23,335	163.6	143.9	4358
15 phr	0.628	202.8	157.6	2,568,287	44.2	46.3	640,604
	5.024	394.7	245.2	580,843	71.8	72.9	127,817
	94.2	816.7	421.9	61,281	133.3	126.9	12,269
	314	978.1	332.7	20,663	137.2	117.8	3617

**Table 3:** Mechanical properties of the vulcanized composites: tear resistance (TES) stress at 100% (SE100), 200% (SE200) and 300% (SE300) of elongation, tensile strength (TS), elongation at break (Eb) and shore hardness, scale D.

Parameter	Reference	Peroxide	10 phr	15 phr
TES (N/mm)	22 ± 2	12 ± 1	18 ± 1	16 ± 3
SE100 (MPa)	3.2 ± 0.1	4.5 ± 0.4	2.6 ± 0.1	2.1 ± 0.1
SE200 (MPa)	3.4 ± 0.1	-	2.8 ± 0.1	2.3 ± 0.1
SE300 (MPa)	3.7 ± 0.1	-	3.0 ± 0.3	2.5 ± 0.6
TS (MPa)	4.7 ± 0.1	4.7 ± 0.4	3.3 ± 0.3	3.1 ± 0.5
Eb (%)	449 ± 11	148 ± 11	330 ± 30	345 ± 82
Hardness (°ShD)	22 ± 1	21 ± 1	19 ± 1	16 ± 1

**Table 4:** Flammability parameters: time to ignition (t_i_), time to flameout (t_o_), heat release rate peak (HRR_p_), heat release rate mean value (HRR_m_), time to HRR_p_ (t_HRR_), HRR_p_/t_HRR_ ratio, total heat release (THR), effective heat of combustion peak (EHC_p_), effective heat of combustion mean value (EHC_m_), mass loss rate peak (MLR_p_), mass loss rate mean value (MLR_m_) and mass loss (m_l_).

Parameter	Reference	Peroxide	10 phr	15 phr
t_i_ (s)	133	77	92	87
t_o_ (s)	446	370	386	356
HRR_p_ (kW/m)	112.9	183.9	192.1	162.7
HRR_m_ (kW/m)	35.6	100.8	104.9	97.7
t_HRR (s)_	210	190	195	195
HRRp/t_HRR_ (kW/ms)	0.54	0.97	0.99	0.83
THR (MJ/m^2^)	12.8	29.8	31.3	26.6
EHC_p_ (MJ/kg)	74.5	72.9	78.7	77.1
EHC_m_ (MJ/kg)	10.7	25.3	26.2	24.0
MLR_p_ (g/s)	0.180	0.118	0.120	0.123
MLR_m_ (g/s)	0.029	0.035	0.035	0.036
MARHE (kW/ms)	0.13	0.26	0.24	0.26
m_l_ (%)	24.6	24.5	24.7	24.6

**Table 5:** Compression strength of the ceramized composites studied.

Name of the Composite	Average Maximum Force (N)		
	1100 °C	950 °C	550–1000 °C
Reference	222 ± 32	214 ± 40	223 ± 73
Peroxide	153 ± 47	258 ± 73	240 ± 49
10 phr	123 ± 42	214 ± 40	226 ± 40
15 phr	130 ± 32	223 ± 73	395 ± 114

## Data Availability

The data presented in this study are available on request from the corresponding author.
